# Systemic Triple Therapy in Metastatic Hormone-Sensitive Prostate Cancer (mHSPC): Ready for Prime Time or Still to Be Explored?

**DOI:** 10.3390/cancers14010008

**Published:** 2021-12-21

**Authors:** Christian Thomas, Martin Baunacke, Holger H. H. Erb, Susanne Füssel, Kati Erdmann, Juliane Putz, Angelika Borkowetz

**Affiliations:** Department of Urology, University Hospital Carl Gustav Carus Dresden, Technische Universität Dresden, 01307 Dresden, Germany; martin.baunacke@uniklinikum-dresden.de (M.B.); holger.erb@uniklinikum-dresden.de (H.H.H.E.); susanne.fuessel@uniklinikum-dresden.de (S.F.); kati.erdmann@uniklinikum-dresden.de (K.E.); juliane.putz@uniklinikum-dresden.de (J.P.); angelika.borkowetz@uniklinikum-dresden.de (A.B.)

**Keywords:** hormone-sensitive prostate cancer, systemic therapy, novel hormonal therapy, taxanes, triple therapy

## Abstract

**Simple Summary:**

Currently, a combination therapy of standard androgen deprivation therapy (ADT) plus docetaxel or androgen receptor-axis-targeted therapy is the gold standard for metastatic hormone-sensitive prostate cancer (mHSPC) treatment. Compared to ADT monotherapy, combination treatment prolongs overall survival by at least 18 months. The latest data has shown that triple treatment with standard ADT plus docetaxel plus abiraterone is superior to standard ADT plus docetaxel. Ongoing clinical trials are investigating triple therapy protocols by affecting diverse signaling pathways that are pivotal in prostate cancer. In this review, we will explore if triple therapy has the potential to be the new standard for mHSPC treatment in the near future.

**Abstract:**

For decades, mono androgen deprivation therapy (ADT) has been the gold standard for metastatic hormone-sensitive prostate cancer (mHSPC) treatment. Several studies have been published within the last seven years demonstrating a significant survival advantage by combination treatment with standard ADT plus docetaxel or androgen receptor-axis-targeted therapy (ARAT) compared to ADT monotherapy. As a result, overall survival can be prolonged by at least 18 months. Recently published congress data of the PEACE-1 study suggests that in the future, triple therapy might be the new gold standard. In addition to this study, which has shown that triple treatment with standard ADT plus docetaxel plus abiraterone is superior to standard ADT plus docetaxel, several other phase III triple therapy studies are currently ongoing. The different modes of action that are investigated reach from AR-targeting over mitotic inhibition and immunotherapy to PARP and AKT inhibition. In this review we will explore if triple therapy has the potential to be the new standard for mHSPC treatment in the near future.

## 1. Introduction

For many decades, single androgen deprivation therapy (ADT) with luteinizing hormone-releasing hormone (LHRH) agonists or LHRH antagonists was the standard treatment for patients with metastatic hormone-sensitive prostate cancer (mHSPC). Acceptable side effects and an antitumoral response in more than 90% of patients combined with easy handling by subcutaneous application led to its broadly and positively accepted application worldwide [1,2,3]. However, despite high initial response rates, the majority of men inevitably progressed to castration-resistant state within 1–3 years. While ADT induces apoptosis in subpopulations of prostate cancer, a certain number of tumor cells survive and usually recur with an androgen-independent phenotype [4,5,6]. Progression to castration-resistant state is a complex process by which cells acquire the ability to both survive and proliferate in the absence of androgens, and this involves variable combinations of clonal selection, adaptive upregulation of anti-apoptotic genes, and alternative growth factor pathways. Frequently, promiscuous reactivation of the AR by growth factors and cytokines can be found [6,7,8,9,10,11,12,13,14,15]. Since the mid-1980s, several attempts have been made to intensify and prolong the efficacy of ADT. Treating 87 mHSPC patients with ADT plus the first-generation antiandrogen flutamide, continuing response and survival at 2 years was 91% [16]. Despite these promising initial results, prospectively randomized, double-blind, placebo-controlled trials failed to show a significant improvement by adding flutamide to standard ADT [17]. The largest study investigating the additional effect of flutamide, including 1387 patients, was published by Eisenberger and colleagues in 1998 and found an increased rate of diarrhea and anemia caused by the first-generation antiandrogen but no impact on overall survival [18]. In 1996, the Casodex Combination Study Group published prospective trial data investigating the effect of additional bicalutamide vs. flutamide on castration in patients with mHSPC. There was no overall survival difference between both groups, and the bicalutamide combination had less common side effects [19]. In summary, adding first-generation antiandrogens to standard ADT may improve overall survival in patients with advanced prostate cancer by 3–5% and 1–2% after 5 and 10 years, respectively [20,21,22]. Based on this sobering data, there was an urgent need for innovative treatment options in advanced prostate cancer. Novel insights in targeting androgen-receptor (AR) and AR-independent pathways have led to the development of promising new compounds at the beginning of the 21st century [23,24,25,26].

After showing their highly potent antitumoral effects in castration-resistant state, the effect of these compounds has been extensively investigated in combination with standard ADT in mHSPC patients over the last decade [27]. In the following, we will present the data of published combination therapy studies that have revolutionized systemic treatment in mHSPC in daily clinical practice. Moreover, we will discuss the options of intensifying systemic treatment by triple therapy approaches targeting the same or different cell signaling pathways.

## 2. Current Options for Modern Systemic Combination Therapy in mHSPC

So far, four novel compounds have been tested for combination therapy in mHSPC: the taxane docetaxel, the cytochrome P17-inhibitor abiraterone, and the two second-generation antiandrogens, enzalutamide and apalutamide (see Table 1). Regarding docetaxel combination therapy, several studies have been published in the past. The first attempt was made by the GETUG15 study group, which could not show a statistically significant survival benefit for the chemohormonal therapy. At least, the hazard ratio (HR) of 0.88 (*p*-value 0.35) showed a modest benefit for adding docetaxel to standard ADT [28].

The CHAARTED study initiated the new area of combination treatment for mHSPC. In total, 790 patients were randomized to either standard ADT with 6 cycles of docetaxel (75 mg/m^2^ every three weeks) or ADT alone. Combining docetaxel with standard ADT significantly improved overall survival by a median of 10.4 months compared to standard ADT (HR 0.72; 95% CI, 0.59–0.89; *p* = 0.0018) [29,38]. Confirmed by the significant survival advantage in the M1 population of the STAMPEDE C study (median benefit of 15.0 months; HR = 0.76; 95% CI 0.62–0.92; *p* = 0.005), docetaxel plus ADT became the new standard for mHSPC patients in the year 2015 [30]. Post hoc analysis of the GETUG15 and CHAARTED study revealed that patients with primary mHSPC and high tumor burden benefitted the most from chemohormonal therapy [39].

Abiraterone is the only CYP17 inhibitor that has been approved so far for the treatment of mHSPC. In the LATITUDE study, patients with “high-risk” mHSPC, defined as the presence of 2 of 3 risk factors (visceral metastases, ≥3 osseous metastases, Gleason score ≥ 8) were included. Addition of 1000 mg/daily abiraterone (plus 5 mg prednisone) resulted in an average survival advantage of 16.8 months compared to standard ADT (HR 0.66; 95% CI 0.56–0.78; *p* < 0.0001) [31,32]. Improved survival by adding abiraterone was confirmed by the STAMPEDE G study (HR 0.61; 95% CI 0.49–0.79; *p* < 0.001) [33,34].

Currently, two second-generation antiandrogens are used for mHSPC combination treatment in daily clinical practice: enzalutamide and apalutamide. In the ARCHES as well as in the ENZAMET study [11], enzalutamide 160 mg/daily was added to standard ADT. For ARCHES, the primary endpoint was radiographic progression-free survival. At initial publication, overall survival data was premature [35]. Just recently, the final analysis was presented at ESMO 2021. With a follow-up of 4 years, 71% of patients in the enzalutamide plus ADT arm compared to 57% of patients in the standard ADT arm were still alive (HR0.66; 95% CI 0.53–0.81; *p* < 0.0001) [36].

In the ENZAMET study, combination treatment with enzalutamide significantly extended overall survival compared to standard ADT. After 3 years’ follow up, 80% of the patients in the enzalutamide arm and 72% of patients in the standard ADT arm were still alive (HR 0.67; 95% CI 0.52–0.86; *p* = 0.002) [37].

The TITAN study is the only phase III trial investigating the impact of adding apalutamide to standard ADT. Apalutamide was administered at 240 mg/daily [40]. At final analysis with 4 years of follow up, 65% of patients in the apalutamide arm and 52% of patients in the control arm were still alive (HR 0.65; CI 0.53–0.79; *p* < 0.0001) [41].

An integral component of the ARCHES, ENZAMET, and TITAN studies was the possibility to include patients with upfront docetaxel administration. The insights into the antitumoral effect of triple therapy for mHSPC and its long-term effect on overall survival will be discussed in the following, particularly in light of the recently presented PEACE-1 study.

## 3. Data Available for Modern Systemic Triple Therapy in mHSPC

So far, four studies have investigated the role of triple therapy in mHSPC: ARCHES, ENZAMET, TITAN, and PEACE-1. Although these studies are not directly comparable due to dosing and timing of docetaxel, they give important insight into the possibility of triple therapy for mHSPC in the future (see Table 2).

In the ARCHES study, 17.8% of patients started docetaxel chemotherapy prior to randomization [35]. Of these, the majority received full six cycles of docetaxel every three weeks. While radiographic progression-free survival was significantly improved by enzalutamide irrespective of prior docetaxel treatment, enzalutamide had no significant impact on overall survival in patients already treated with docetaxel chemotherapy (HR 0.74; CI 0.46–1.20).

In the TITAN study, 10.7% of the patients started docetaxel-treatment before randomization to apalutamide or placebo. The median number of docetaxel cycles was six in both arms. Even here, apalutamide effectively prolonged radiographic progression-free survival but not overall survival (HR 1.12; CI 0.59–2.12) [40,41].

The largest triple therapy study in mHSPC published so far is the ENZAMET trial. Here, 503 of 1125 men (45%) received docetaxel chemotherapy [37]. Of these, docetaxel was given in 178 patients (35%) within 12 weeks before randomization. Therewith, the ENZAMET study represents a mixture of prior and concurrent triple therapy regimens. The full planned six cycles of docetaxel were given to 159 of 243 patients (65%) in the enzalutamide arm and 181 of 238 (76%) in the standard arm. In concordance with the ARCHES and TITAN study, overall survival was not significantly prolonged by addition of enzalutamide in patients receiving docetaxel treatment (HR 0.90; CI 0.62–1.31). Irrespectively, PSA progression-free survival and clinical progression-free survival improved significantly by adding enzalutamide in patients already receiving docetaxel (HR 0.46; CI 0.36–0.60 and HR 0.48; CI 0.37–0.62, respectively).

Until recently, all published triple therapy studies have failed to show an overall survival improvement compared to combination therapy. However, the overall survival data of the PEACE-1 study presented in September 2021 at the ESMO congress may bring new hope for future triple therapy in mHSPC [42]. PEACE stands for Prostate Cancer Consortium in Europe, and it is an academic European program that aims to conduct phase 3 trials for men with prostate cancer. Analogous to the STAMPEDE trial, PEACE has a flexible structure enabling the inclusion of novel treatment modalities. Between 2013 and 2018, 1173 patients with mHSPC have been randomized in the PEACE-1 study. Patients were primary metastatic and presented at least one bone lesion. Randomized to four arms in a 1:1:1:1 ratio, patients received standard of care (SOC), SOC + abiraterone, SOC + radiotherapy, or SOC + abiraterone + radiotherapy. The fact that docetaxel was permitted as part of SOC in 2015 (and mandatory since 2017) provided the basis for investigating the impact of adding androgen receptor-axis-targeted therapy (ARAT) to chemohormonal therapy. In total, 355 patients received SOC plus docetaxel (−/+ radiotherapy of the prostate), and 355 received SOC plus docetaxel plus abiraterone (−/+ radiotherapy of the prostate). The median number of docetaxel cycles was six in each arm.

Adding abiraterone to ADT plus docetaxel significantly improved overall survival (HR 0.75; CI 0.59–0.95; *p* = 0.017). Especially patients with high-volume disease seem to benefit from triple therapy (HR 0.72; Ci 0.55–0.95; *p* = 0.019). The effect is markedly less pronounced in patients with low-volume disease (HR 0.83; CI 0.5–1.38; *p* = 0.66). Interestingly, radiotherapy of the prostate did not seem to affect overall survival in the subgroup analysis. The benefit of triple-therapy with abiraterone was present in patients receiving and not receiving local radiotherapy (HR 0.76; CI 0.53–1.07 and HR 0.73; CI 0.52–1.03, respectively).

(https://www.urotoday.com/conference-highlights/esmo-2021/esmo-2021-prostate-cancer/132237-esmo-2021-a-phase-3-trial-with-a-2x2-factorial-design-in-men-with-de-novo-metastatic-castration-sensitive-prostate-cancer-overall-survival-with-abiraterone-acetate-plus-prednisone-in-peace-1.html (accessed on 10 December 2021)).

The question comes up of why improved overall survival was not achieved by triple therapy in the beforementioned trials, ARCHES, ENZAMET, and TITAN. Currently, this question cannot be answered unambiguously. Although the existing data on triple therapy is inconsistent, a closer look at the studies’ discrepancies might give some comprehensible explanations. In the PEACE-1 study, all patients included in the study had primary metastatic disease, whereas in ARCHES, ENZAMET, and TITAN, this was verifiable only in 66%, 61%, and 81%, respectively. As patients with metachronous metastases tend to have an improved outcomes compared to primary metastatic disease, the effect of triple therapy might not be as impressive [28]. Another distinctive feature between PEACE-1 versus ARCHES, ENZAMET, and TITAN is the medication intake. While in ARCHES, TITAN, and partially in ENZAMET, chemotherapy was started before ARAT treatment, the use of abiraterone was started concomitantly to docetaxel in the PEACE-1 study. Mechanistically, not only abiraterone but also docetaxel is affecting the AR axis. The direct effect on AR signaling by abiraterone results not only from stopping conversion of adrenal steroids to androgens but also from inhibition of intratumoral de novo steroidogenesis [43]. Docetaxel indirectly affects the AR by abrogating its translocation via inhibiting tubulin polymerization [44]. From a mechanistic point of view, the complementary mechanisms of both drugs on the AR axis might be pronounced when they are started concomitantly. In addition, the different mechanisms of action in the ARAT group have to be discussed. A central antitumoral function of second-generation antiandrogens is the inhibition of AR translocation [45]. When AR translocation is already impaired by previous docetaxel treatment, second-generation antiandrogens might not be able to show their full antitumoral potential.

Based on the network analysis of Feyerabend and colleagues, ADT + ARAT tends to be more effective than ADT plus docetaxel [46]. With the potential option of triple therapy in the future, the role of solely chemohormonal therapy has to be discussed critically. Maybe in the future, docetaxel treatment in mHSPC will just play a role in a triple therapy setting or in patients with histopathological partial dedifferentiated prostate cancer.

## 4. Ongoing Studies Investigating the Role of Triple Therapy in mHSPC

Several registration trials are currently investigating the role of triple therapy in mHSPC. The different modes of action that are investigated reach from AR targeting over mitotic inhibition and immunotherapy to PARP and AKT inhibition (see Table 3).

Regarding intensified AR targeting, one of the most promising trials is the ARASENS study. This phase III study will consist of approximately 1300 mHSPC patients who will be randomized in a 1:1 ratio to receive 1200 mg/daily of the second-generation antiandrogen darolutamide daily compared to control, each in addition to standard ADT and docetaxel. Subjects will be stratified at randomization by tumor volume and by alkaline phosphatase levels. The primary endpoint is overall survival. The estimated study completion date will be in May 2022.

(https://clinicaltrials.gov/ct2/show/NCT02799602?term=ARASENS&cond=prostate+cancer&draw=2&rank=1 (accessed on 17 November 2021)).

Immune modulation has revolutionized the treatment of renal and bladder cancer in the last five years. In prostate cancer as well, several studies have been launched with a focus on checkpoint inhibition. However, no PD1/PDL1 or CTLA4 inhibitor has been approved for the treatment of prostate cancer so far [47]. Currently, no phase III data is available for treatment with checkpoint inhibitors in mHSPC. The ongoing MK3475-991 study investigates the efficacy and safety of pembrolizumab plus enzalutamide plus ADT versus placebo plus enzalutamide plus ADT in mHSPC patients (*n* = 1232). Primary outcome measures are radiographic progression-free survival and overall survival. The estimated study primary completion date will be in September 2026.

(https://clinicaltrials.gov/ct2/show/NCT04191096?term=991&cond=prostate+cancer&draw=1&rank=1 (accessed on 17 November 2021)).

The PI3K/AKT pathway plays a pivotal role in the progression of solid tumors. In prostate cancer, this pathway interacts with the AR axis. As a result, single inhibition of one of these pathways results in a reciprocal feedback loop. Translational studies have shown that combining an AKT inhibitor with an AR inhibitor leads to a prolonged disease stabilization in mCRPC [48]. Capivasertib is a potent PI3K/AKT inhibitor and has actually been tested in three pivotal trials with breast and prostate cancers. The ProCAID trial, a phase II study, tested capivasertib in combination with docetaxel in patients with metastatic castration-resistant prostate cancer (mCRPC). Although clinical progression-free survival was not significantly improved in the capivasertib arm, there was an impressive overall survival benefit for patients treated with this potent PI3K/AKT inhibitor (31.2 months vs. 20.3 months, respectively) [49]. Currently, the CAPItello-281 study is assessing the efficacy and safety of capivasertib plus abiraterone plus ADT versus abiraterone plus ADT in participants with mHSPC (*n* = 1000). In this phase III study, patients will be characterized by PTEN deficiency. The estimated study primary completion date will be in November 2025.

(https://clinicaltrials.gov/ct2/show/NCT04493853?term=capitello&cond=Prostate+Cancer&draw=2&rank=1 (accessed on 17 November 2021)).

Talazoparib is a novel PARP inhibitor approved to treat BRCA-mutated, HER2-negative, locally advanced or metastatic breast cancer patients. In prostate cancer, the TALAPRO-3 study investigates the effect of adding talazoparib to enzalutamide plus ADT vs. enzalutamide plus ADT in mHSPC patients with DNA damage repair (DDR) gene mutation. Approximately 550 patients will be randomized. The estimated study primary completion date will be in April 2027.

(https://clinicaltrials.gov/ct2/show/NCT04821622?term=talapro&cond=Prostate+Cancer&draw=2&rank=1 (accessed on 17 November 2021)).

The AMPLITUDE study represents a comparable trial. Here, the promising PARP inhibitor niraparib is used in combination with abiraterone plus ADT versus abiraterone plus ADT in patients with DDR-gene-mutated mHSPC. In total, 788 patients will be included. The estimated study primary completion date will be in May 2027.

(https://clinicaltrials.gov/ct2/show/NCT04497844?term=amplitude&cond=Prostate+Cancer&draw=2&rank=1 (accessed on 17 November 2021)).

## 5. Final Remarks and Future Perspectives

Currently, combination therapy of ADT plus docetaxel or ARAT is the new gold standard in the treatment of patients with mHSPC. Compared to ADT monotreatment, combination therapy has significantly improved oncologic outcomes. These findings have led to ongoing investigations to further optimize treatment in mHSPC. According to the principle “the more, the better”, several triple therapy studies have been launched within the last years. The positive results of the recently demonstrated PEACE-1 study take a favorable view on triple therapy in mHSPC. However, is triple therapy ready for prime time? Based on the available preliminary data, no unrestricted recommendation can actually be given to implement triple therapy for mHSPC in daily clinical practice. However, the results of the ARASENS trial, which are expected in the first half of 2022, might change this view. In the future, it has to be investigated which modes of action have to be combined to obtain the best antitumoral efficacy. According to currently released data, intensified hormonal treatment by adding ARAT plus taxane chemotherapy to standard ADT is the most promising triplet. Moreover, pharmacological targeting of programmed cell death, PI3K/AKT signaling, or PARP has shown promising preliminary data, and phase III studies are ongoing to evaluate their role for triple therapy in mHSPC. Particularly due to the numerous drugs that are potentially available for combination treatment of mHSPC, a multidrug adaptive therapy to maintain a controllable stable tumor burden seems to be a promising approach in the future to further prolong tumor response and thereby survival [50].

## 6. Conclusions

In conclusion, systematic combination treatment has dramatically changed the treatment landscape in mHSPC within the last six years. While currently, combination treatment by adding ARAT or docetaxel is the therapy standard for the majority of patients, mHSPC treatment options will further expand in the next years. According to the maxim “hit hard, hit early”, most likely we will discuss very soon the options of triple therapy and maybe quadruple therapy in the future.

## Figures and Tables

**Table 1:** Combination therapy studies in mHSPC.

Study	Intervention	Prior Local Therapy (%)	Docetaxel Use (%)	Initial Serum PSA (ng/mL)	Median Follow-Up (Months)	Overall Survival Benefit (HR; 95% CI)
GETUG-15 [26]	ADT plus docetaxel 75 mg/m^2^ every three weeks for 6 cycles vs. ADT	28	−/−	26	84	0.88; 0.68–1.14
CHAARTED [27,28]	ADT plus docetaxel 75 mg/m^2^ every three weeks for 6 cycles vs. ADT	37	−/−	51	54	0.72; 0.59–0.89
STAMPEDE C [29] (M1 subgroup)	ADT plus docetaxel 75 mg/m^2^ every three weeks for 6 cycles vs. ADT	−/−	−/−	−/−	43	0.76; 0.62–0.92
LATITUDE [30]	ADT plus abiraterone 1 g/prednisone 5 mg daily vs. ADT	0	−/−	−/−	52	0.66; 0.56–0.78
STAMPEDE G [31,32] (M1 subgroup)	ADT plus abiraterone 1 g/prednisone 5 mg daily vs. ADT	6	−/−	−/−	34	0.61; 0.49–0.79
ARCHES [33,34]	ADT plus enzalutamid 160 mg daily vs. ADT	27	18	5	45	0.66; 0.53–0.81
ENZAMET [35]	ADT plus enzalutamid 160mg daily vs. ADT	39	45	−/−	34	0.67; 0.52–0.86
TITAN [36,37]	ADT plus apalutamide 240 mg daily vs. ADT	16	11	−/−	44	0.65; 0.53–0.79

ADT = androgen deprivation therapy, CI = confidence interval, HR = hazard ratio.

**Table 2:** Available subgroup data for triple therapy with ADT + docetaxel as standard of care in mHSPC.

Study	Patients Receiving Triple Therapy (*n*)	Start of Docetaxel Application to NHT	Docetaxel Cycles	Effect of NHT on OS HR; 95% CI
ARCHES [33,34]	205	Prior	Full 6 cycles administered in 86% of patients	0.74; 0.46–1.20
ENZAMET [35]	503	Prior (35%) and concomitant (65%)	Full 6 cycles administered in 71% of patients	0.90; 0.62–1.31
TITAN [36,37]	58	Prior	In median, 6 cycles administered	1.12; 0.59–2.12
PEACE-1 [40]	710	Concomitant	Full 6 cycles administered in 100% of patients	0.75; 0.59–0.95

ADT = androgen deprivation therapy; HR = hazard ratio; CI = confidence interval; OS = overall survival.

**Table 3:** Ongoing phase III studies for triple therapy in mHSPC.

Study	Mode of Action	Treatment Arms	Patient Population (*n*)	Primary Endpoint	Estimated Study Completion
ARASENS	Intensified AR axis plus microtubule centrosome targeting	Darolutamide 600 mg/daily + docetaxel 75 mg/m^2^ every three weeks for 6 cycles + ADT vs. docetaxel 75 mg/m^2^ every three weeks for 6 cycles + ADT	1300	Overall survival	5/2022
MK3475-991	Intensified targeting of AR axis plus PD1 inhibition	Pembrolizumab 200 mg every three weeks + enzalutamide 160 mg/daily + ADT vs. enzalutamide 160 mg/daily + ADT	1232	Radiographic progression-free survival and overall survival	9/2026
CAPItello-281	Intensified targeting of AR axis plus inhibition of PI3K/AKT pathway	Capivasertib 800 mg/daily (day 1-4/7) + abiraterone 1000 mg + prednisone 5 mg + ADT vs. abiraterone 1000 mg + prednisone 5 mg + ADT	1000	Radiographic progression-free survival	11/2025
TALAPRO-3	Intensified targeting of AR axis plus DNA damage repair inhibition	Talazoparib 0.5 mg + enzalutamide 160 mg/daily + ADT vs. enzalutamide 160 mg/daily + ADT	550	Radiographic progression-free survival	4/2027
AMPLITUDE	Intensified targeting of AR axis plus DNA damage repair inhibition	Niraparib 200 mg/daily + abiraterone 1000 mg + prednisone 5 mg + ADT vs. abiraterone 1000 mg + prednisone 5 mg + ADT	788	Radiographic progression-free survival	5/2027

ADT = androgen deprivation therapy, AR = androgen receptor, PD = programmed death, PI3K/AKT = proteinkinase B.
